# Treatment of chronic mini-thoracotomy wound pain and lung herniation with intercostal cryoablation and surgical mesh repair: a case report

**DOI:** 10.1186/s13019-024-02864-y

**Published:** 2024-06-21

**Authors:** Eun Yeung Jung, Seok Soo Lee

**Affiliations:** Department of Thoracic and Cardiovascular Surgery, Yeungnam University College of Medicine, 170 Hyeonchung-ro, Nam-gu, Daegu, 42415 Korea

**Keywords:** Minimally invasive surgery, Thoracotomy, Neuralgia, Intercostal cryoablation, Surgical mesh

## Abstract

**Background:**

The incidence of minimally invasive heart surgery via mini-thoracotomy (MT; right anterior thoracotomy) is on the rise, accompanied by an increase in post-MT intercostal nerve neuralgia and the risk of lung herniation through the incision site. While various methods have been proposed to address these issues, none have been commonly effective. In this case report, we attempted to simultaneously address these problems by performing intercostal cryoablation (IC) and mesh repair.

**Case presentation:**

A 43-year-old male was referred to our hospital for chronic post-thoracotomy neuralgia following heart surgery via MT, involving patch closure of an atrial septal defect and tricuspid annuloplasty. He presented with intercostal nerve neuralgia and lung herniation accompanied by severe pain. Despite medication and lidocaine injections, there was no relief. Consequently, he underwent surgical treatment with IC for chronic MT wound pain and simultaneously underwent mesh repair for a lung hernia. He was discharged from hospital free of complications. Subsequently, he no longer required further pain medication and experienced a favorable recovery.

**Conclusion:**

Our findings suggest that concurrent IC and mesh repair can effectively relieve chronic post-MT intercostal nerve neuralgia and severe lung herniation pain in patients who underwent MT surgery, leading to a decrease in opioid medication usage.

## Background

Mini-thoracotomy (MT) has been advocated as an alternative approach to heart surgery without sternotomy. However, MT is associated with severe post-thoracotomy intercostal neuralgia (PTN), which can lead to respiratory complications such as hypoventilation, hypoxia, atelectasis, pulmonary infections, and respiratory failure [1, 2]. The methods for treating PTN include oral medication, intravenous opioids, intercostal nerve blocks, and epidural analgesia. However, these methods have a short duration of action and may provoke complications such as respiratory depression, neurotoxicity, and epidural hematoma. Intercostal cryoablation (IC) is also known to be effective in managing intercostal neuralgia [3]. It has resulted in reduced opioid requirements and postoperative visual analogue pain scores (VAS) [4]. While this method has traditionally been used for relieving pain in patients who underwent a Nuss procedure or those with rib fractures [5, 6], its benefit has rarely been demonstrated in patients underwent MT. Another potential complication that may occur after MT is lung herniation through the incision site [7]. Frequently associated symptoms with this condition include pain, persistent cough, shortness of breath and hemoptysis, nevertheless, many of these hernias can be completely asymptomatic [8]. If this causes symptoms such as pain or lung injury, surgical approach may be necessary. We present a case report of the post-MT patient with chronic PTN and intercostal lung herniation treated by IC and mesh repair.

## Case presentation

**Fig. 1:**
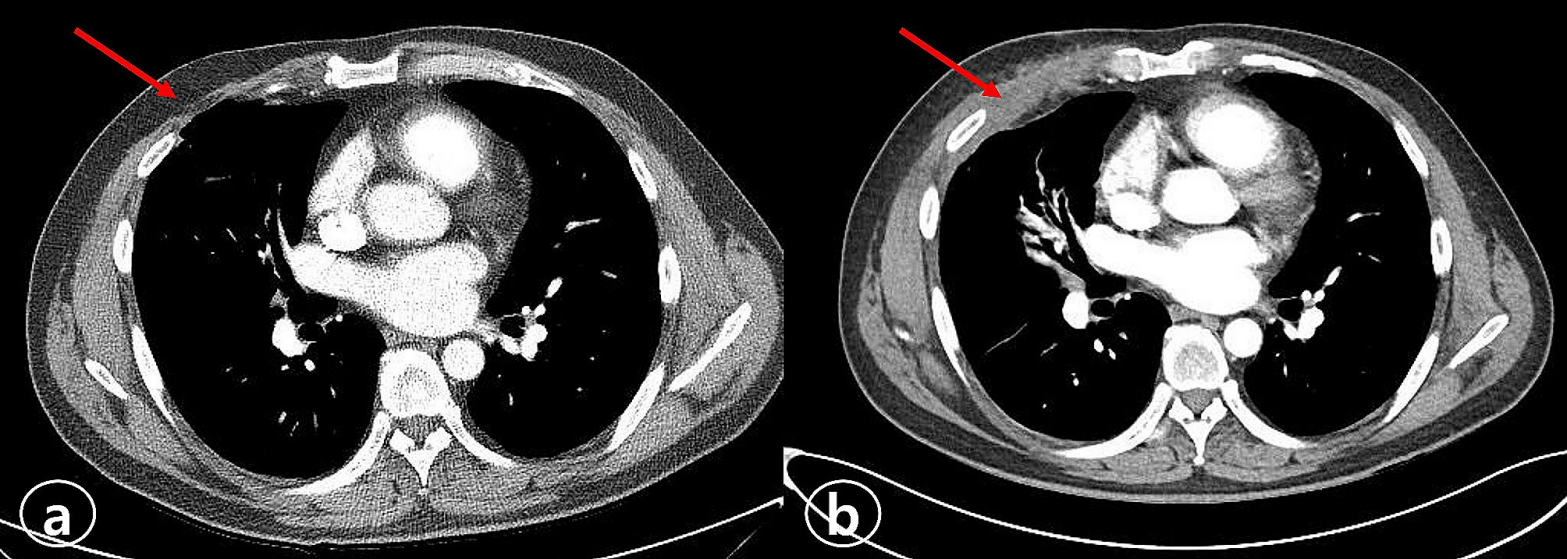
Preoperative and Postoperative CT scans (**a**) Preoperative chest computed tomography (CT) shows an intercostal lung hernia through the 4th intercostal space (arrow). (**b**) Postoperative chest CT demonstrates the disappearance of the intercostal lung herniation (arrow)

**Fig. 2:**
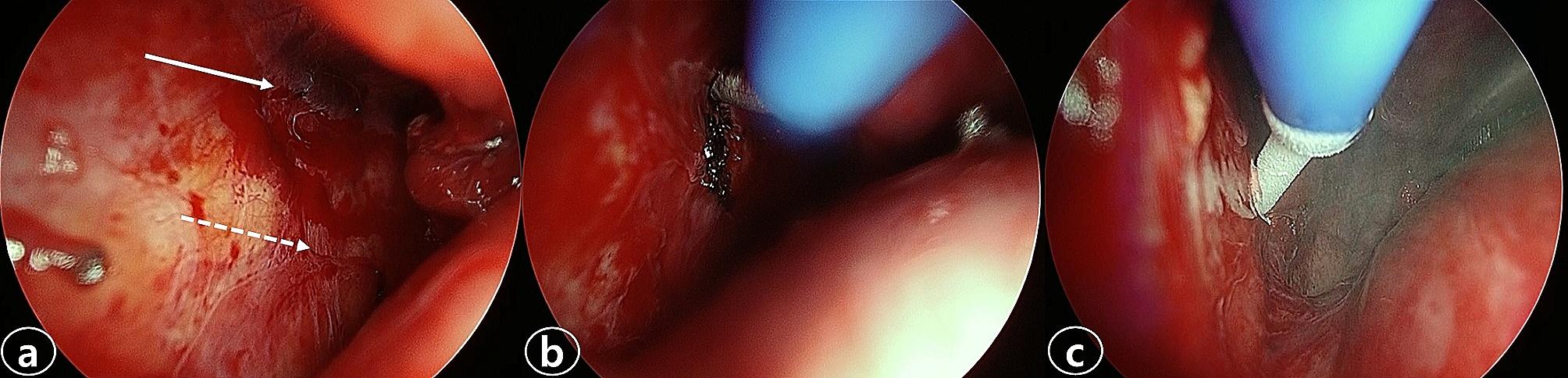
Intercostal cryoablation (**a**) The intercostal nerves of the 4th (arrow) and 5th (dash arrow) ribs were identified by video-assisted thoracoscopy. (**b**,**c**) Intercostal cryoablation was performed using a cryoanalgesia probe approximately 4 cm lateral to the spine. The probe was applied directly to the neurovascular bundle at − 60 °C for 120 s per intercostal nerve

A 43-year-old male was referred to the General Thoracic Surgery (GTS) department in May 2023 for chronic PTN, which was provoked after heart surgery in November 2021. He had a surgical history of patch closure of an atrial septal defect and tricuspid annuloplasty surgery via MT 1 year ago in the Cardiovascular Surgery department. His computed tomography (CT) scan before the heart surgery revealed no chronic lung disease or other underlying conditions. His postoperative course was uneventful, and he was discharged on the 14th postoperative day after heart surgery. Six months later, he visited the hospital for pain at the MT site. His VAS score was 8 out of 10. There was no sign of redness or infection at the thoracotomy wound. A physical examination revealed tenderness at the level of the 4th and 5th ribs. Laboratory values were normal, and a chest X-ray showed no abnormalities. He was prescribed nonsteroidal anti-inflammatory drug (NSAID) medications and went back. After 4 months, he visited again because the pain at the MT site had not disappeared. Lidocaine was injected at the 4th intercostal space (ICS), and he was prescribed opioids and neuralgia analgesics. He was also recommended to visit a pain clinic. In May 2023, after 18 months post-heart surgery, he visited the GTS department for chronic pain at the operation site. He complained that it was not relieved and was getting worse. The chest CT revealed an intercostal lung hernia through the 4th ICS (Fig. 1A). We planned the surgical treatment for pain relief by IC and mesh repair of lung hernia. To address the lung herniation, we reopened the previous surgical incision site (4th ICS) and encountered difficulty in obtaining a clear field of view for precise intercostal cryoablation solely through this site. Therefore, we utilized video-assisted thoracoscopy, accessing through the 7th ICS (1 cm insicion, troca insertion), to enhance visibility. The intercostal nerves of the 4th and 5th ribs were identified by thoracoscopy. Employing a cryoanalgesia probe (CRYO2, AtriCure, Mason, OH, USA), we conducted cryoablation in the areas corresponding to the 4th and 5th intercostal nerve, approximately 4 cm lateral to the spine (see Fig. 2A). The probe was applied directly to the neurovascular bundle at -60 °C for 120 s per intercostal nerve (see Fig. 2B). Subsequently, we repaired the pleural space using Bard Mesh (Davol, Inc., Warwick, RI, USA), and closed the intercostal spaces with Vicryl #1 − 0 suture. Before the wound close, a 16 Fr. chest tube was inserted at the thoracoscope insertion site to conclude the procedure, with no additional incisions made. The chest tube was removed on the following day without complications. On the 2nd postoperative day, the patient no longer required opioid medication. His chest pain was rated 2 out of 10. He had no difficulty with breathing or sensory. He was discharged on the 7th postoperative day with a prescription for NSAIDs to be taken as needed for pain. He was visited on the 14th postoperative day. The patient reported his pain level as 3 out of 10, but he had no other discomfort. He did not require any medication for pain and received a prescription just in case. At his 8-week follow-up, the patient reported his pain level remained at 2 out of 10, and he no longer required medication. A follow-up chest CT revealed the disappearance of the intercostal lung herniation (Fig. 1B). He still had no tingling or paresthesia.

## Discussion

MT has been advocated as an alternative approach to sternotomy for heart surgery. This method offers advantages such as reduced bone bleeding and increased thoracic stabilization. However, it also has some disadvantages. The duration of anesthesia and surgery can be prolonged due to technical difficulties, and the detection of accidental trouble can be delayed because of the limited surgical field of view [9]. These issues can be improved with increased surgeon experience. However, problems such as severe PTN, provoked by the MT incision, present another challenge that must be overcome, as it can lead to respiratory complications or other morbidity. Recently, with the increasing number of minimally invasive surgery via MT, there has been a growing interest in PTN control. IC is considered one of the viable treatments for PTN management. Cryoablation has traditionally been used under various conditions for the treatment of rib fractures, Nuss operation, and post-full thoracotomy pain. This approach has led to decreased opioid usage and postoperative visual analogue pain scores (VAS) [4]. Bauman et al., in a recent retrospective study, demonstrated reduced hospital length of stay (LOS), diminished need for narcotics, and consequent cost savings [10]. This method is considered an effective means to alleviate PTN and has shown positive results in this case patient. The disadvantage of IC lies in its invasiveness compared to alternative methods such as medication or percutaneous injection. Consequently, if the objective is solely pain management, there is the added complexity of administering treatment under general anesthesia. Moreover, nerves regenerate at a rate of approximately 3 mm/day along the remaining perineural structures, eventually restoring normal sensation [10, 11]. Nevertheless, excessive nerve damage can result in numbness [12, 13]. It’s worth noting that no specific side effects were identified in this case patient. However, it’s essential to remain mindful of these limitations when employing IC.

After surgery via MT, other potential side effects may occur due to thoracotomy, aside from PTN, including pulmonary hernia, rib fracture, and intercostal hemorrhage [14, 15]. Surgeons and patients must always consider these complications alongside PTN. Controversy persists regarding the necessity of surgical repair for intercostal hernias, as they typically do not present a significant threat unless complications such as incarceration and strangulation occur, leading to symptoms such as hemoptysis and pain. While both surgical and conservative approaches are advocated, spontaneous recovery of lung herniation is improbable [15]. Therefore, in cases where lung herniation poses a risk to the patient’s well-being (e.g., hemoptysis, decreased oxygen saturation, severe pain), surgical intervention may be warranted. In this case, the patient presented with chronic PTN and a lung hernia after MT surgery. Despite opioid medication and local anesthetic injections, the pain persisted and even increased. For such patients, surgical IC treatment and repair of the hernia can be a viable option. It has been shown to provide excellent analgesia along with decreased hospital length of stay and reduced use of narcotics, thereby improving the patient’s quality of life. Our findings indicate that concurrent IC and mesh repair can effectively relieve chronic PTN pain in MT patients and lead to a reduction in opioid medications.

## Data Availability

The data is stored in our database and can be provided at any time upon request.

## Abbreviations

CT: Computer Tomography
VATS: Video-Assistant Thoracic Surgery
MT: Mini-Thoracotomy
IC: Intercostal Cryoablation
PTN: Post-Thoracotomy Intercostal Neuralgia
VAS: Visual Analogue Pain Score
GTS: General Thoracic Surgery
CT: Computed Tomography
NSAID: Nonsteroidal Anti-Inflammatory Drug
ICS: Intercostal Space
